# Supplementary material for: Structure of the M. tuberculosis DnaK−GrpE complex reveals how key DnaK roles are controlled
Source: Nat Commun. 2024 Jan 22;15:660. doi: 10.1038/s41467-024-44933-9 (PMC10803776; doi:10.1038/s41467-024-44933-9)
Supplement: Supplementary file 1 — Supplementary Information [file 41467_2024_44933_MOESM1_ESM.pdf]

Supplementary Information

Structure of the *M. tuberculosis* DnaK-GrpE complex reveals how key DnaK roles are controlled

Authors:

Xiansha Xiao[1], Allison Fay[2], Pablo Santos Molina[2], Amanda Kovach[1],
Michael S Glickman[2] and Huilin Li[1]*

Affiliations:

[1]Department of Structural Biology, Van Andel Institute, Grand Rapids, MI, USA
[2]Immunology Program, Sloan Kettering Institute, New York, NY, USA

*Correspondence should be addressed to H.L. (Huilin.Li@vai.org)

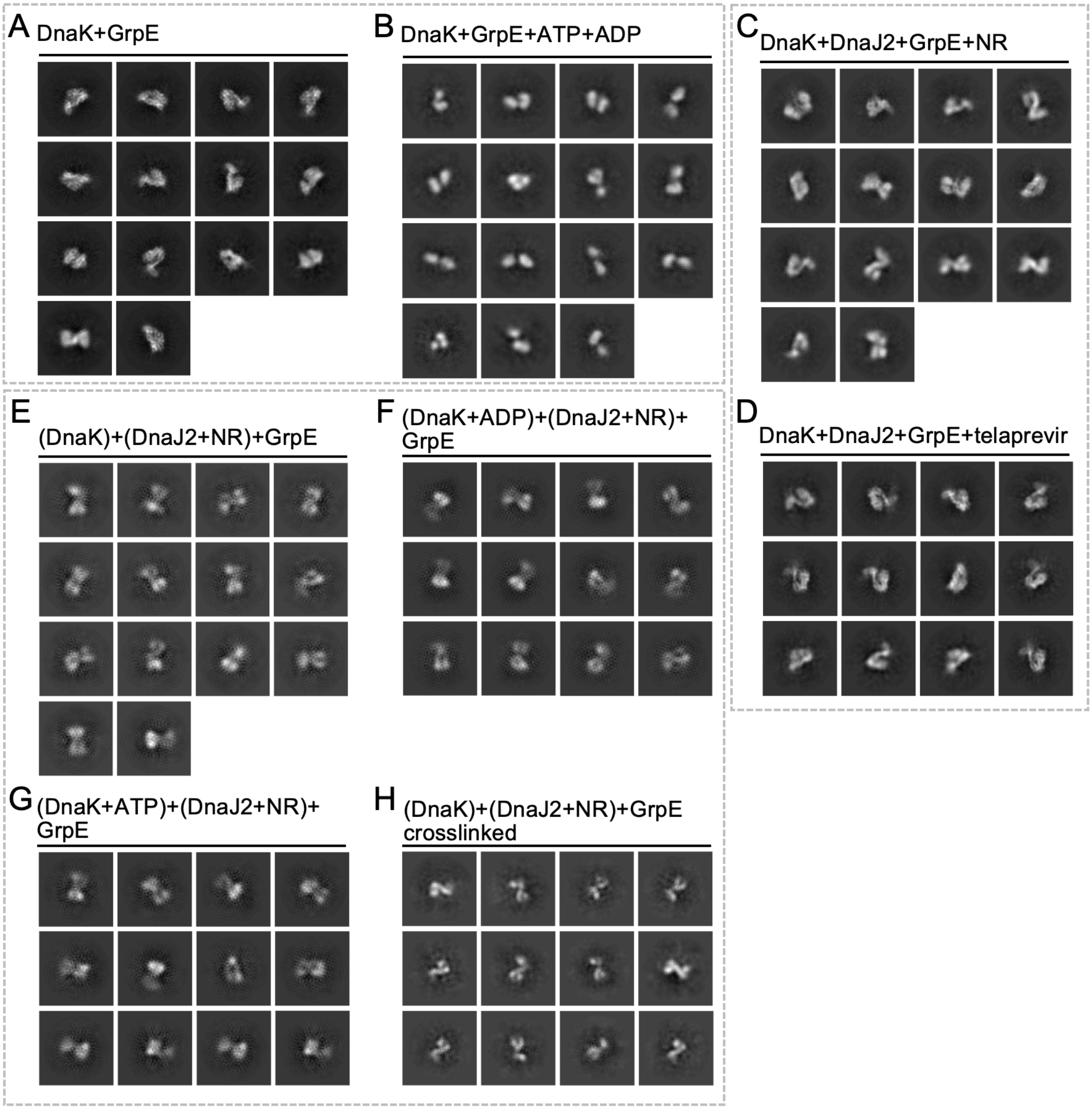

Supplementary Figure 1. Cryo-EM analyses of in vitro complex assembly under various conditions. A) 2D class averages of the mixture of as-purified DnaK and GrpE show the formation of the DnaK–GrpE complex. **B**) 2D class averages of the mixture of DnaK and GrpE in the presence of 0.1 mM ATP and 0.1 mM ADP. No DnaK–GrpE complex was observed. **C-D**) 2D class averages of the mixture of DnaK, GrpE, and DnaJ2, with either the substrate peptide NRLLLTG (C) or the peptide inhibitor telaprevir (D). Only the binary DnaK–GrpE complex, but not the ternary DnaK–GrpE–DnaJ complex, was observed. **E-G**) 2D class averages of the mixtures of DnaK preincubated with no nucleotide (E), 0.1 mM ADP (F), and 0.1 mM ATP (G) + 10 mM $MgCl_2$, followed by the addition of DnaJ2 preincubated with the substrate peptide, and finally the addition of GrpE. **H**) 2D class averages of the cross-linked samples that was assembled otherwise as in (E).

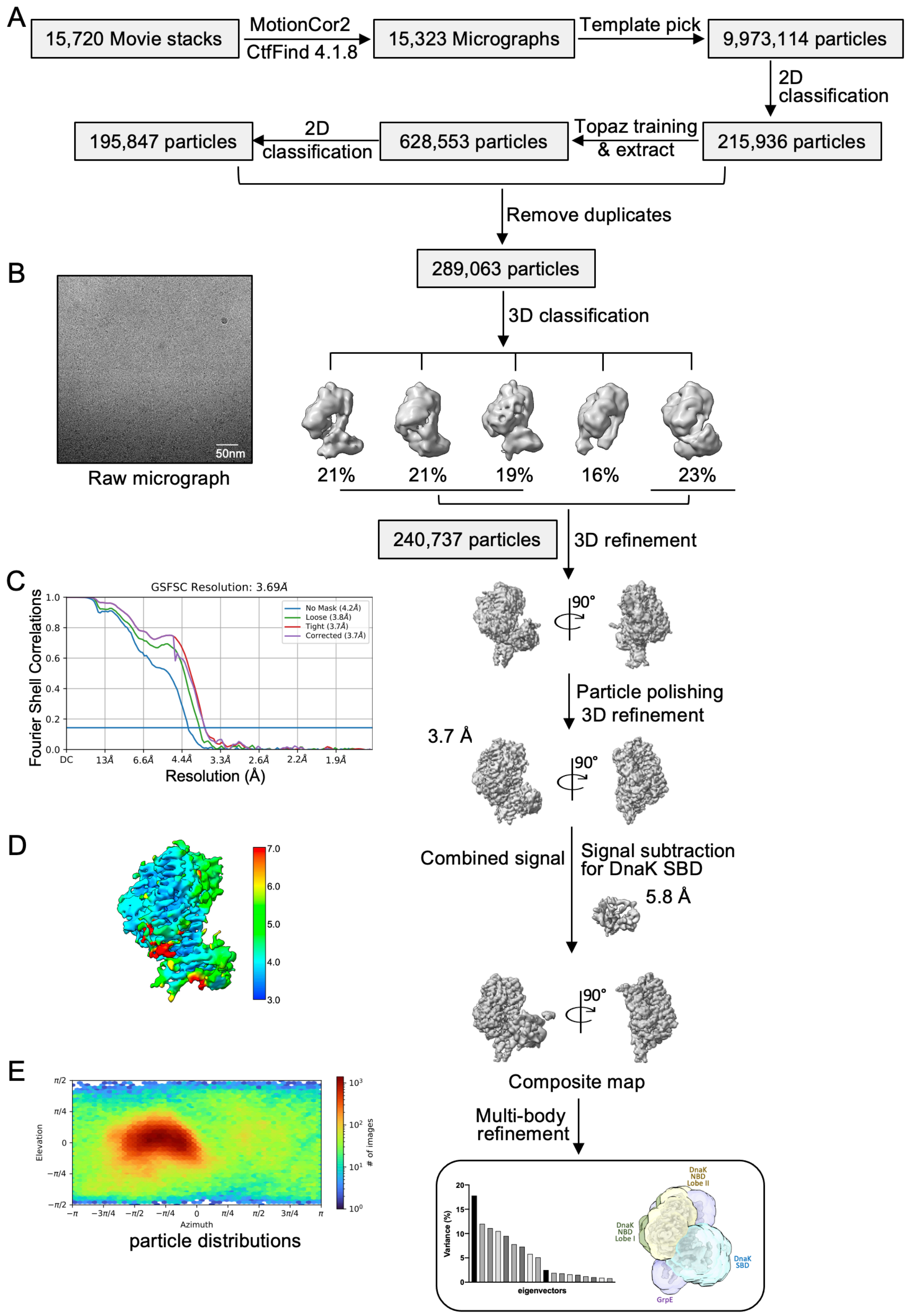

Supplementary Figure 2. Workflow of image processing and 3D reconstruction of cryo-EM images of the Mtb DnaK-GrpE complex. A) 2D classification was performed twice to remove the isolated DnaK and GrpE particles. The DnaK SBD was poorly resolved in the overall refined 3.7 Å map (green). Through masking and focused refinement, the EM density in the partially flexible DnaK SBD region was improved (orange). **B**) A typical raw micrograph selected from 15,323 micrographs collected. **C**) Gold standard Fourier shell correlation (FSC) of the EM map with or without various masks as indicated. **D**) The EM map of the DnaK-GrpE complex surface rendered and colored by the local resolution estimation. **E**) Eulerian angle distribution of the DnaK-GrpE particles used in the final 3D reconstruction.

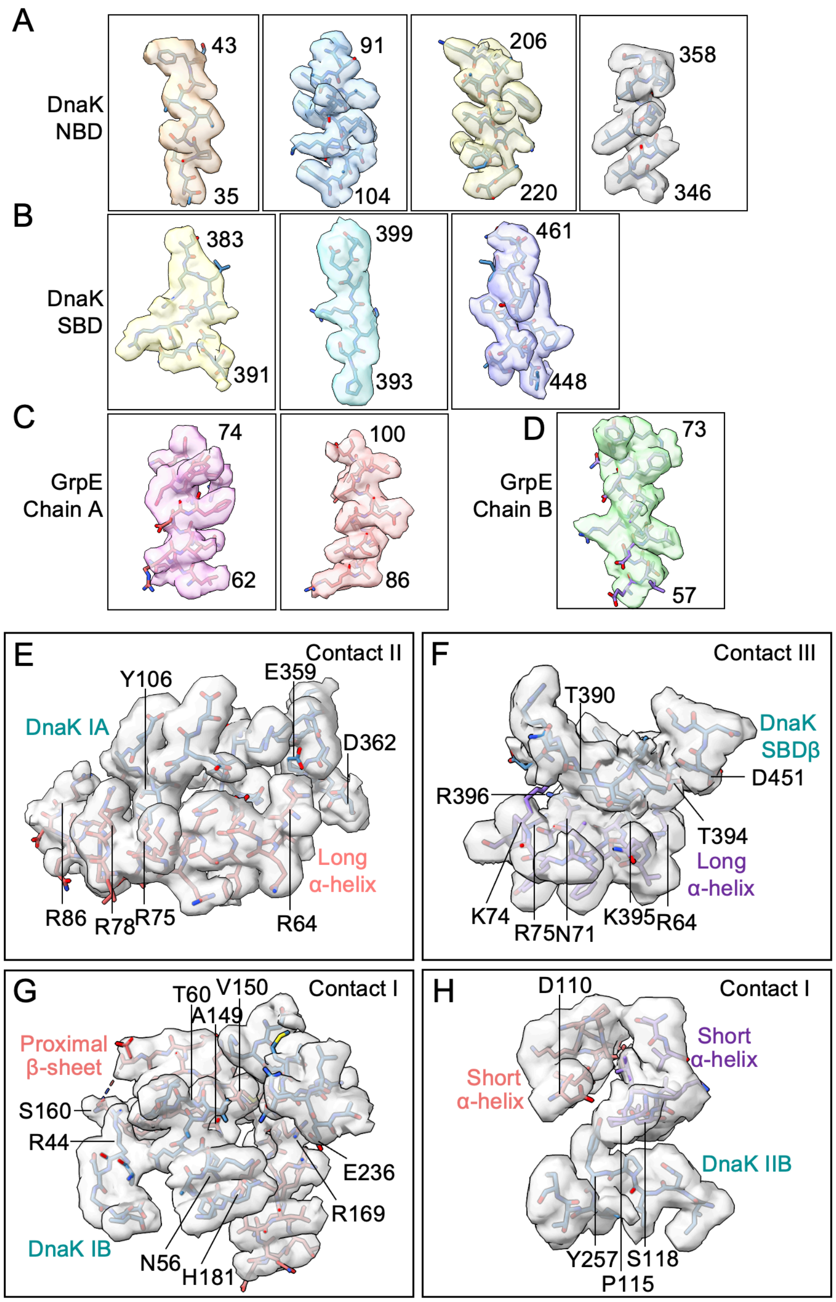

Supplementary Figure 3. Superimposition of the EM map and atomic model of the DnaK–GrpE complex in selected regions. A-D) Selected regions in DnaK-NBD (A), DnaK-SBD (B), and in GrpE molecule A (C) and GrpE molecule B (D). **E-H**) Superimposition of map and structure model at DnaK and GrpE contact regions II (E), III (F), and I (G, H). The model is shown in stick representation and the map in transparent surfaces.

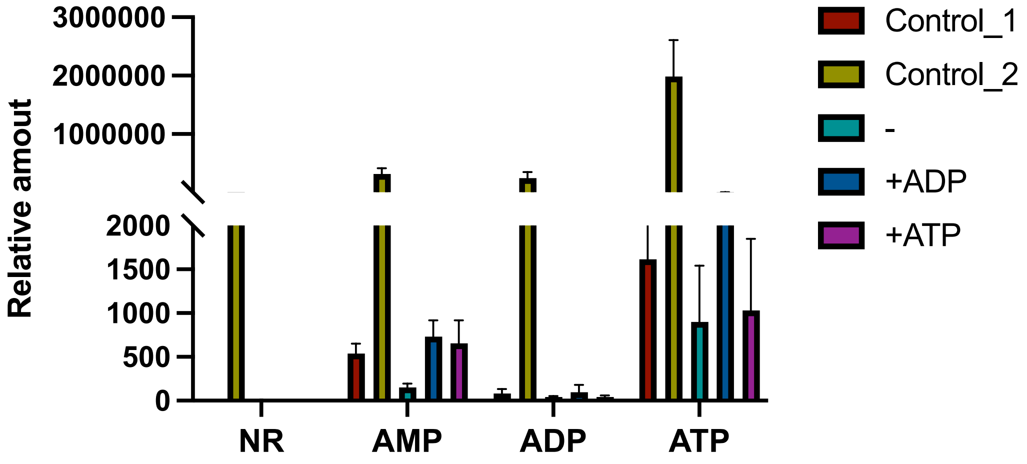

Supplementary Figure 4. Mass spectrometry indicates an absence of peptide (NRLLLTG) and nucleotides (AMP, ADP, and ATP) in the in vitro assembled DnaK-GrpE complex used in the current study. For sample preparation, 10 mM DnaK bound either to no nucleotide (-, cyan), or to 100 mM ADP (+ADP, blue), or to 100 mM ATP (+ATP, purple) was mixed with 10 mM DnaJ2 that was pre-incubated with 100 mM peptide substrate (NRLLLTG, NR) and incubated for 30 min. 10 mM GrpE was added and further incubated for 10 min. The unbound peptide and nucleotide were removed by gel filtration chromatography. Peak fractions corresponding to the DnaK-GrpE complex were collected and concentrated for metabolite extraction by organic solvent. And the extracts were analyzed by mass spectrometry. Each experiment was repeated four times except for the negative controls (Control_1, red) and positive controls (Control_2, yellow) which were repeated two times. Positive controls used extract from a sample containing 10 mM DnaK-GrpE-DnaJ2, 100 mM each of NR, ADP, AMP, and ATP without running gel filtration. The negative controls used the gel filtration buffer containing neither nucleotide nor peptide substrate (25 mM HEPES pH7.5, 150 mM KCl, 2 mM DTT). Source data are provided as a Source Data file.

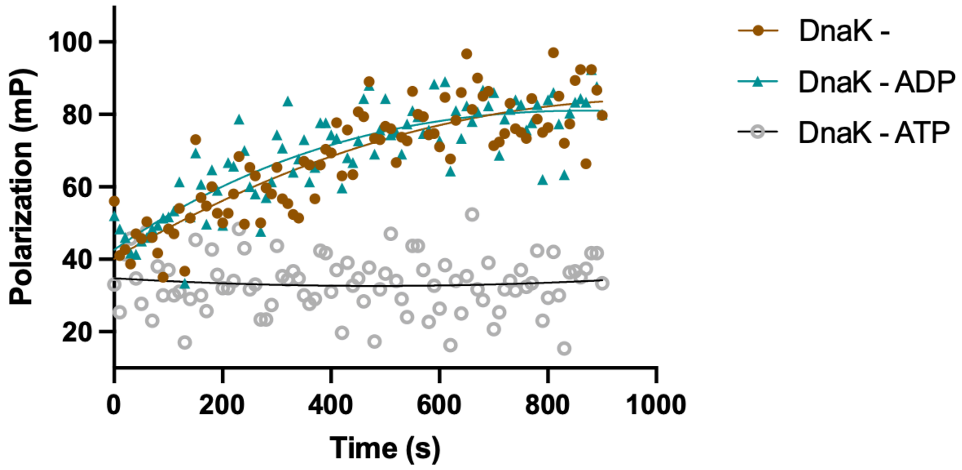

Supplementary Figure 5. Fluorescence polarization (FP) experiments of DnaK in the presence or absence of nucleotides. The affinity of the Mtb DnaK for the substrate peptide was high in the presence of ADP or in the absence of any nucleotide. ATP apparently blocked the binding of DnaK and substrate peptide. Source data are provided as a Source Data file.

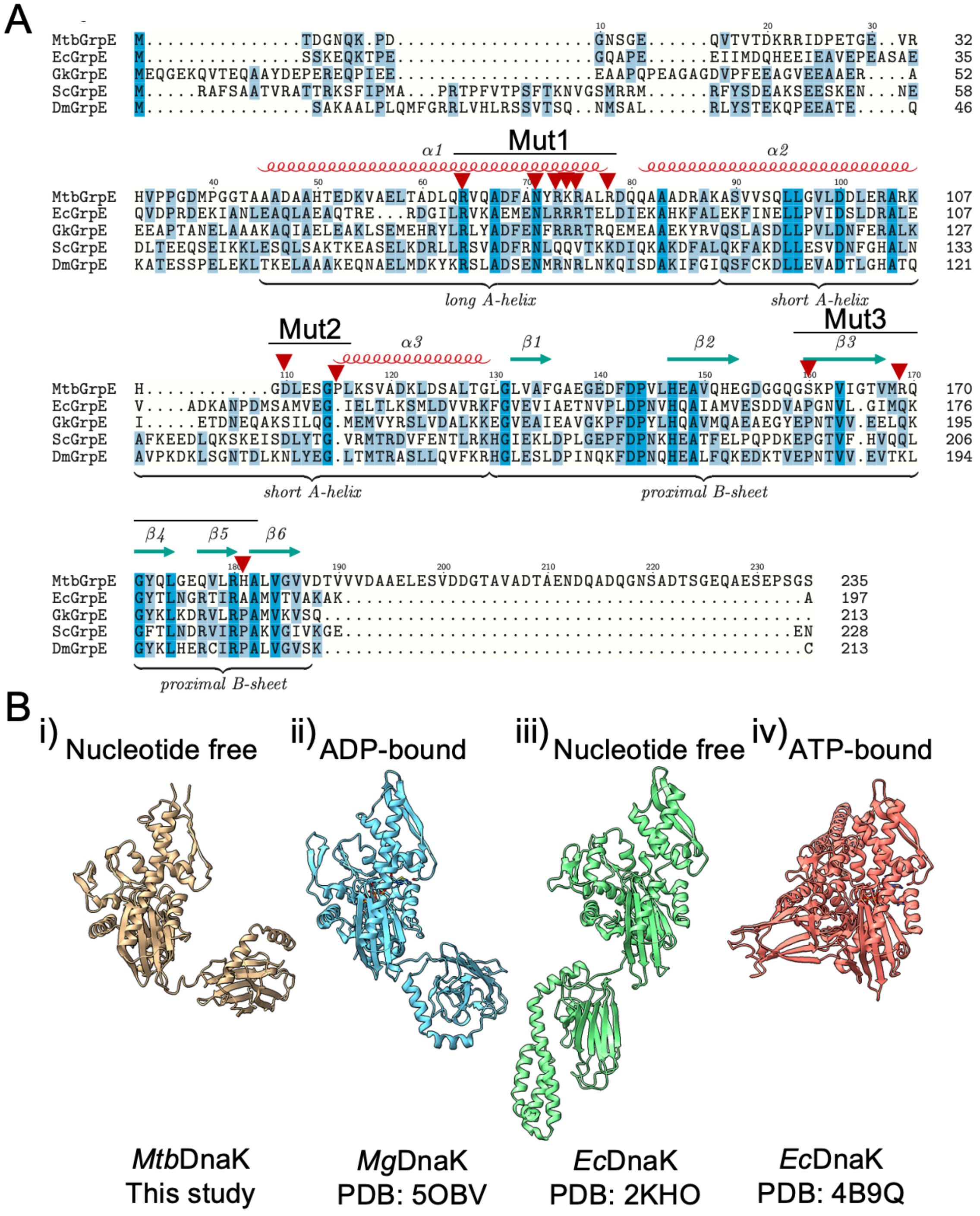

Supplementary Figure 6. Sequence and structural comparisons among GrpE homologs. A) Sequence alignment of the cellular GrpE from Mtb (accession number P9WMT5), *E. coli* (accession number B7NSB2)*,* and *G. kaustophilus* (accession number Q5KWZ6) and the mitochondrial GrpE from *Saccharomyces cerevisiae* (accession number P38523) and *Drosophila melanogaster* (accession number P48604). The red arrowheads point to residues mutated in this study. **B**) Structural comparison of Mtb DnaK of this study with the ADP-bound *M. genitalium* DnaK (PDB ID 5OBV), nucleotide free *E. coli* DnaK (PDB ID 2KHO) and ATP-bound *E. coli* DnaK (PDB ID 4B9Q). The structures were aligned by their respective NBDs but shown separately for clarity.

A

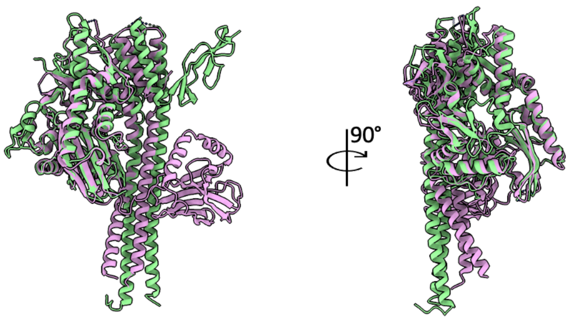

B

DnaK-GrpE	PDB: 1DKG	RMSD
chain D	chain D	1.7
chain E	chain A	3.3
chain F	chain B	3.1
Overall		3.9

C

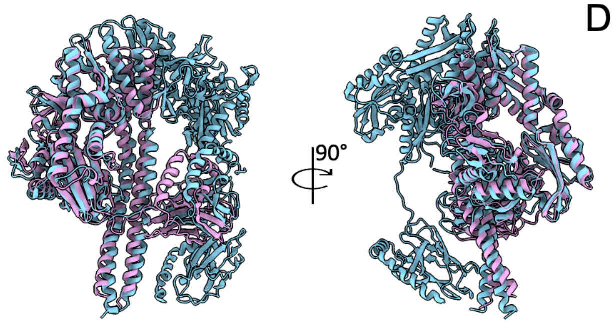

D

DnaK-GrpE	PDB: 4ANI	RMSD
chain D	chain C	3.5
chain E	chain A	2.3
chain F	chain B	2.9
Overall		5.3

Supplementary Figure 7. Superposition of the Mtb DnaK-GrpE structure with two homologue structures. A-B) Superposition of the Mtb and *E. coli* DnaK-GrpE structures (A). The table lists the r.m.s.d. values among the pairs of subunits (B). **C-D**) Superposition of the Mtb and *G. kaustophilus* DnaK-GrpE structures (C). The table lists the r.m.s.d. values among the pairs of subunits. Note that the DnaK SBD was truncated in the *E coli* structure (A-B), and the *G. kaustophilus* DnaK and GrpE formed a 2:2 complex in the crystal structure (C-D).

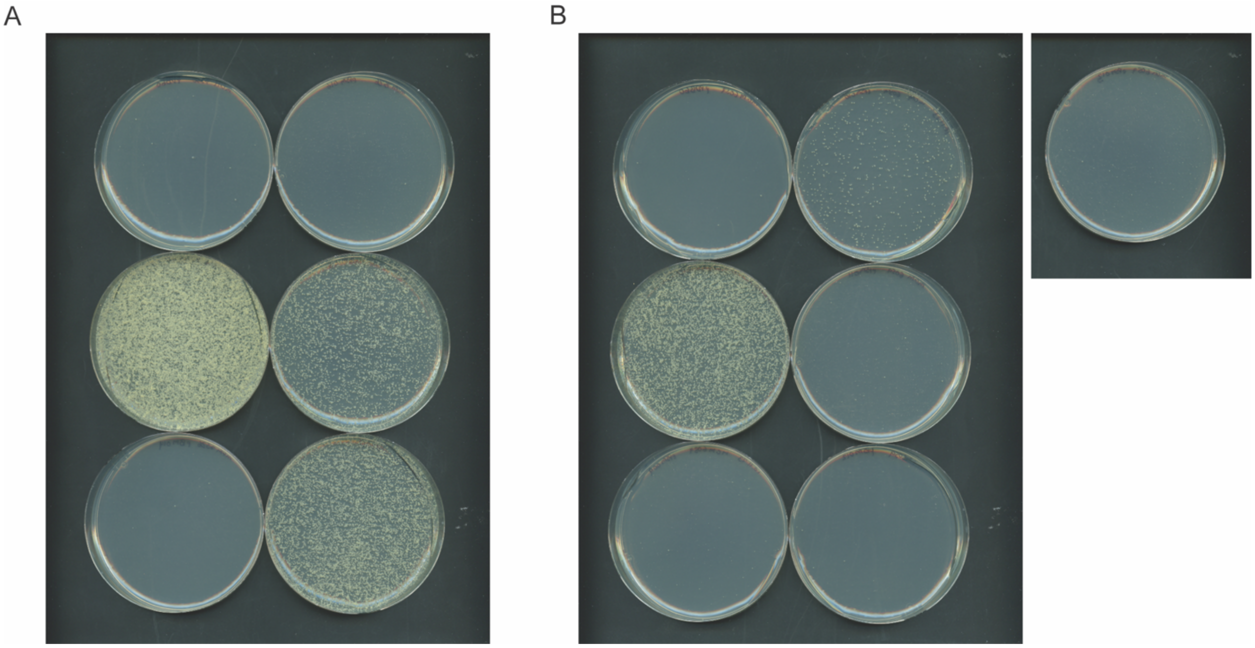

Supplementary Figure 8. Plates of various Msmeg strains produced in this study. A) Strain carrying a *grpE* chromosomal deletion and a second copy of *grpE* encoded at the phage integration site *attB*, (Δ*grpE attB::PdnaK-grpE kan* (MGM7526)) was subjected to marker exchange with streptomycin marked *attB* integrating plasmids encoding the wild type or *grpE* mutated versions of the Mtb DnaK operon. Transformation with pDB60 (empty vector, left, top), pPSM002 (encoding Mtb ALFA-GrpE wt, left middle), pPSM014 (encoding Mtb ALFA-GrpE Mut 1-3, left, bottom), pPSM016 (encoding Mtb ALFA-GrpE Mut-1, right, top), pPSM017 (encoding Mtb ALFA-GrpE Mut-2, right, middle), or pPSM018 (encoding Mtb ALFA-GrpE Mut-3, right, bottom). **B)** Strain carrying chromosomal deletion of *dnaK* and a second copy of *dnaK* encoded at phage integration site *attB* (Δ*dnaK attB::dnaK-twinstrep kan* (MGM6059) was subjected to marker exchange with streptomycin marked attB integrating plasmids encoding the wild type or mutant versions of the Mtb DnaK operon: pDB60 (empty vector, left, top), pPSM001 (encoding Mtb ALFA-DnaK wt, left, middle), pPSM009 (encoding Mtb ALFA-DnaK Mut 2-4, left, bottom), pPSM010 encoding Mtb ALFA-DnaK Mut-1, middle, top), pPSM011 (encoding Mtb ALFA-DnaK Mut-2, middle, middle), pPSM012 (encoding Mtb ALFA-DnaK Mut-4, middle, bottom), or pPSM013 (encoding Mtb ALFA-DnaK Mut-3, right, top).

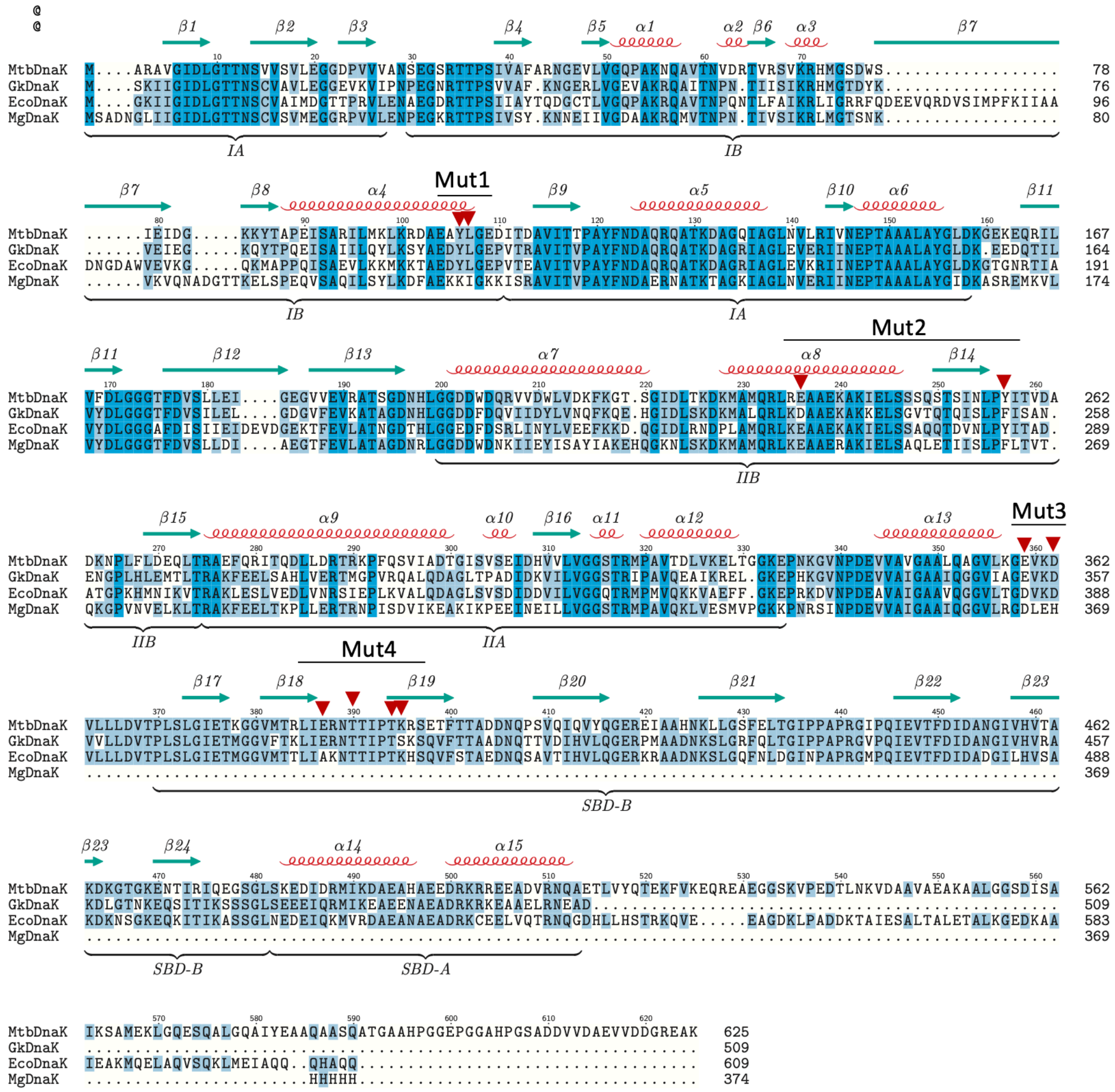

Supplementary Figure 9. Conserved residues in DnaK. Sequence alignment of DnaK from *M. tuberculosis* (accession number P9WMJ9), *G. kaustophilus* (accession number Q5KWZ7), *E. coli* (accession number P0A6Y8), and *M. genitalium* G37 (accession number P47547). The red arrowheads point to residues mutated in this study.

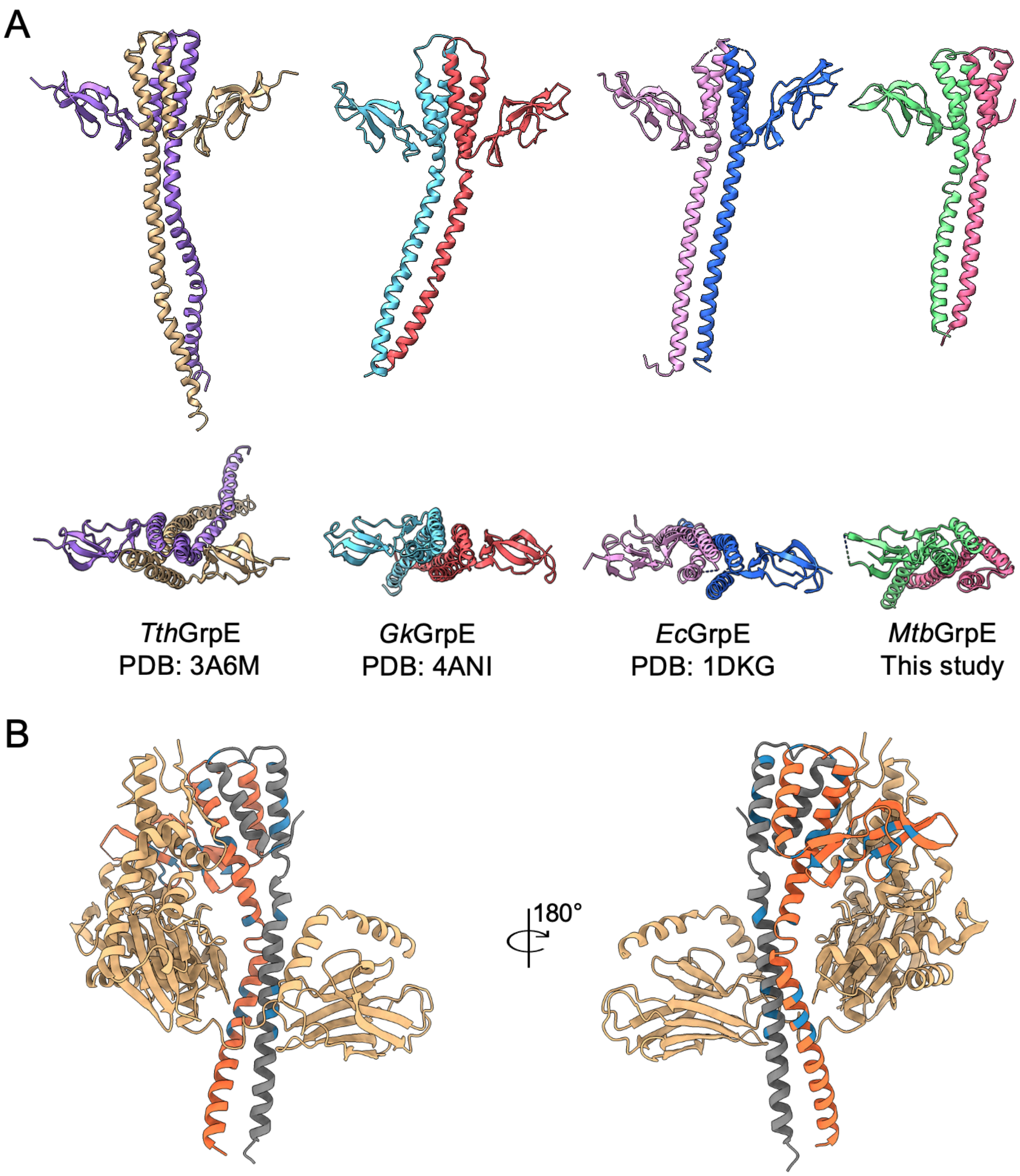

Supplementary Figure 10. Comparison of the available prokaryotic GrpE structures. A) Side and top view of the Mtb GrpE structure (this study) aligned with the *T. thermophilus* GrpE (PDB ID 3A6M), *G. kaustophilus* GrpE (PDB ID 4ANI), and *E. coli* GrpE (PDB ID 1DKG). Despite similar overall architecture, these structures differ in terms of asymmetry and bending of the central coiled coil. **B**) Highly conserved residues (light blue) among the prokaryotic GrpE are mapped to regions that interact with DnaK.

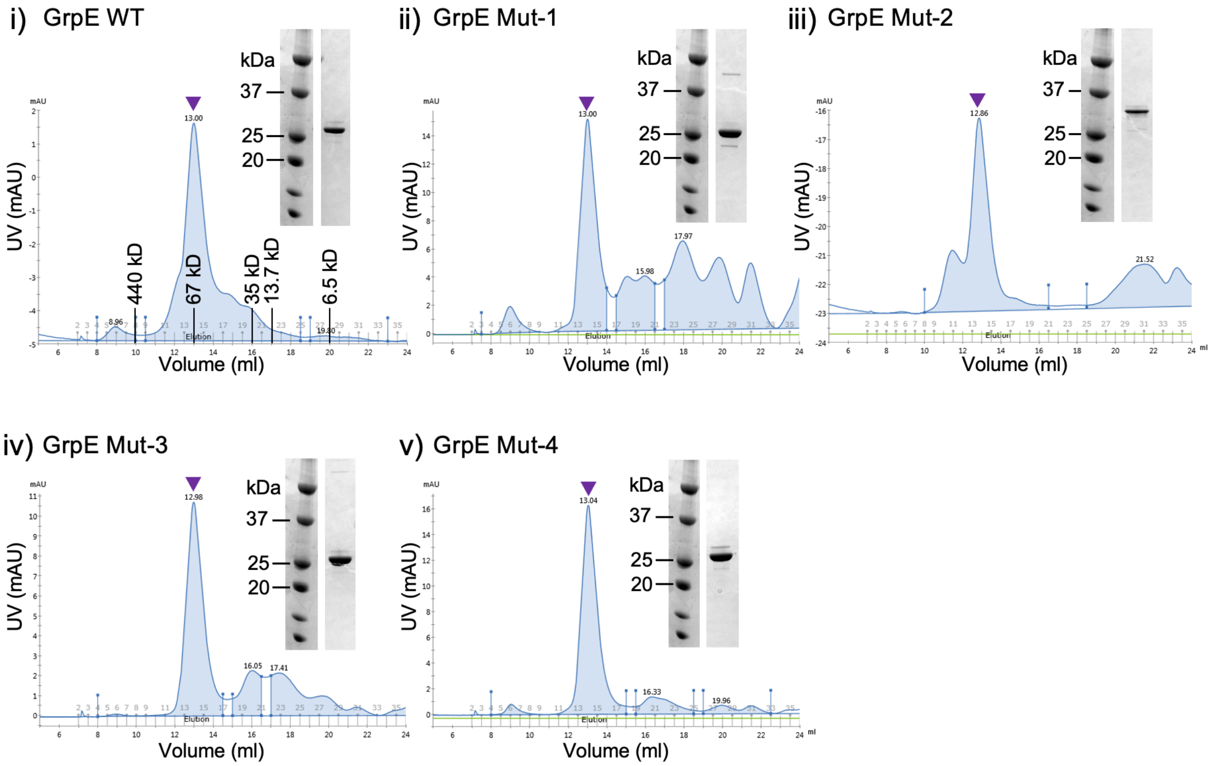

Supplementary Figure 11. Gel filtration profiles of the WT and mutant GrpE. These profiles are from the WT GrpE (i) and Mut-1 to Mut-4 GrpE proteins (ii – v). The elution volumes of molecular standards are marked by vertical lines in the first panel. Coomassie stained SDS-PAGE gel images of the samples eluted at the expected GrpE dimer volumes are inserted as a subpanel in the filtration profiles, and the molecular weight standards (kDa) for each gel image are labeled. The purple arrowhead in each profile marks the dimer elution peak for the WT and the Mut-1 to Mut-4 GrpE proteins. Only peak fractions corresponding to the WT GrpE dimer were collected for all biochemical studies.

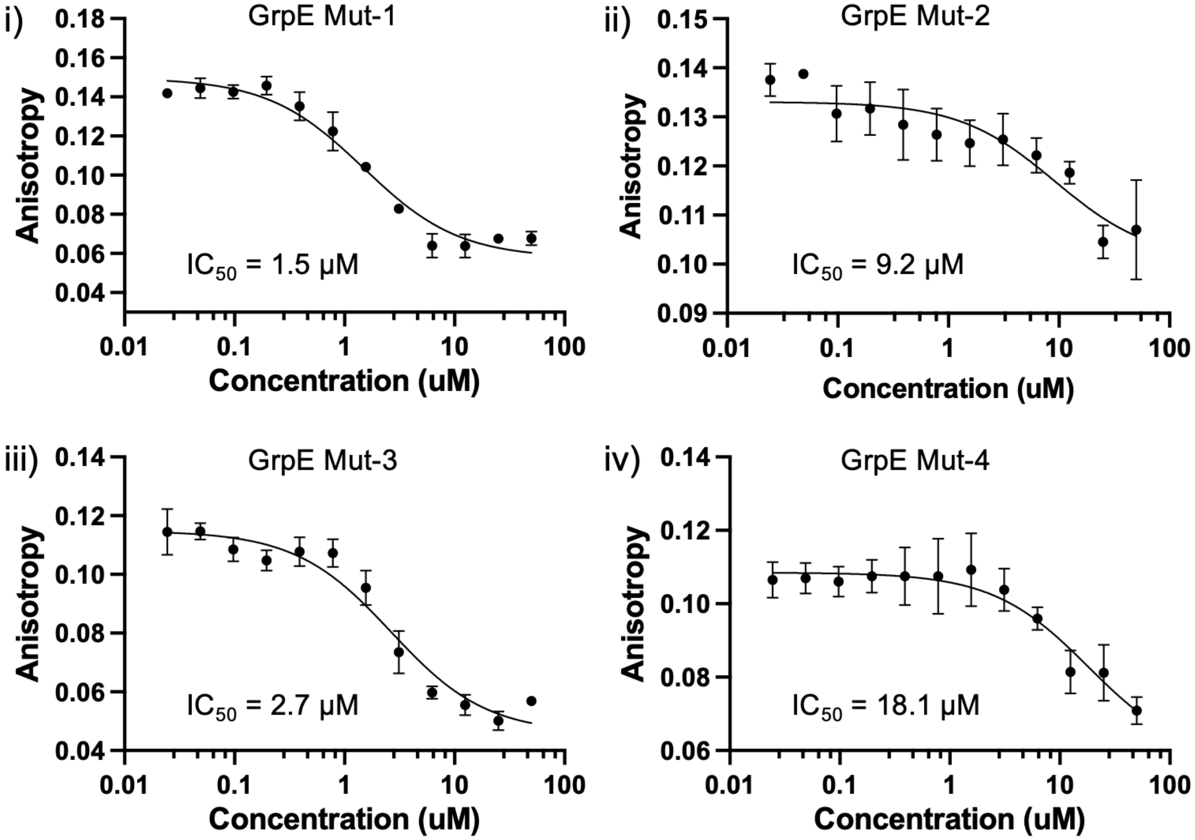

Supplementary Figure 12. Fluorescence polarization of the F-NR bound Mtb DnaK at an increasing concentration of Mut-1 to Mut-4 GrpE proteins. Binding of WT and mutant GrpE facilitated substrate release from DnaK with an IC_{50} of 1.5 µM for GrpE Mut-1 (i), 9.2 µM for GrpE Mut-2 (ii), 2.7 µM for GrpE Mut-3 (iii), and 18.1 µM for GrpE Mut-4 (iv). Source data are provided as a Source Data file.

Supplementary Table 1. Average values with standard deviations of the ITC experiments across the replicates

Protein (cell)	Protein (syringe)	ΔH[a] (kcal/mol)	N[a]	Kd[b] (10^{-6} mol/L)
DnaK	GrpE	-5.63 ± 0.73	0.69 ± 0.16	0.24 ± 0.17
DnaK	GrpE Mut-1	-1.94 ± 0.76	1.01 ± 0.20	1.61 ± 2.31
DnaK	GrpE Mut-2	-4.28 ± 0.25	1.01 ± 0.01	0.71 ± 0.34
DnaK	GrpE Mut-3	-6.11 ± 0.14	0.73 ± 0.06	1.42 ± 0.25
DnaK Mut-1	GrpE	-29.68 ± 29.58	0.13 ± 0.11	1.07 ± 1.21
DnaK Mut-2	GrpE	NB	NB	NB
DnaK Mut-3	GrpE	-7.46 ± 0.04	0.40 ± 0.01	0.18 ± 0.02
DnaK Mut-4	GrpE	-8.27 ± 3.02	0.44 ± 0.02	0.50 ± 0.12
DnaK-ADP	GrpE-ADP	-10.6 ± 2.33	0.69 ± 0.04	1.79 ± 0.81
DnaK-ATP	DnaK-ATP	-80 ± 0.00	0.07 ± 0.03	20.7 ± 8.91

[a] Values were determined from fits of the ITC profile using the single binding site model.
[b] Kd was determined from Ka derived from fits of the ITC profile using the single binding site model.
NB indicates no binding was observed. The errors reflect the standard deviation of the experimental data from the fits of the ITC profile.
