# Supplementary material for: Structure of the M. tuberculosis DnaK−GrpE complex reveals how key DnaK roles are controlled
Source: Nat Commun. 2024 Jan 22;15:660. doi: 10.1038/s41467-024-44933-9 (PMC10803776; doi:10.1038/s41467-024-44933-9)
Supplement: Supplementary file 3 — Description of Additional Supplementary Files [file 41467_2024_44933_MOESM3_ESM.pdf]

Description of Additional Supplementary Files:

Supplementary Data 1: All strains used in this study.

Supplementary Data 2: All plasmids including relevant features used in this study.

Supplementary Data 3: All primers used in this study.

Supplementary Data 4: Cryo-EM data collection, refinement, and validation statistics.

Supplementary Data 5: Transition list. Ion paired LC parameters. MS-QQQ parameters.

Supplementary Movie 1: A large-scale rotation of the GrpE dimer, accompanying a small up/down movement of the DnaK SBD in the Mtb DnaK-GrpE complex.

Supplementary Movie 2: An opening/closing movement of the DnaK NBD lobe I against lobe II, accompanying a large rotation of the GrpE dimer in the Mtb DnaK-GrpE complex.

Supplementary Movie 3: A large opening/closing movement of the DnaK NBD lobe I against lobe II, accompanying a small movement of the GrpE dimer in the Mtb DnaK-GrpE complex.
