# Supplementary material for: Structure of the M. tuberculosis DnaK−GrpE complex reveals how key DnaK roles are controlled
Source: Nat Commun. 2024 Jan 22;15:660. doi: 10.1038/s41467-024-44933-9 (PMC10803776; doi:10.1038/s41467-024-44933-9)
Supplement: Supplementary file 12 — Reporting Summary [file 41467_2024_44933_MOESM12_ESM.pdf]

nature portfolio

Corresponding author(s): Huilin Li

Last updated by author(s): 2023-12-6

Reporting Summary

Nature Portfolio wishes to improve the reproducibility of the work that we publish. This form provides structure for consistency and transparency in reporting. For further information on Nature Portfolio policies, see our Editorial Policies and the Editorial Policy Checklist.

Statistics

For all statistical analyses, confirm that the following items are present in the figure legend, table legend, main text, or Methods section.

n/a	Confirmed	
☐	☒	The exact sample size (*n*) for each experimental group/condition, given as a discrete number and unit of measurement
☐	☒	A statement on whether measurements were taken from distinct samples or whether the same sample was measured repeatedly
☐	☒	The statistical test(s) used AND whether they are one- or two-sided<br>*Only common tests should be described solely by name; describe more complex techniques in the Methods section.*
☒	☐	A description of all covariates tested
☒	☐	A description of any assumptions or corrections, such as tests of normality and adjustment for multiple comparisons
☐	☒	A full description of the statistical parameters including central tendency (e.g. means) or other basic estimates (e.g. regression coefficient) AND variation (e.g. standard deviation) or associated estimates of uncertainty (e.g. confidence intervals)
☐	☒	For null hypothesis testing, the test statistic (e.g. *F*, *t*, *r*) with confidence intervals, effect sizes, degrees of freedom and *P* value noted<br>*Give P values as exact values whenever suitable.*
☒	☐	For Bayesian analysis, information on the choice of priors and Markov chain Monte Carlo settings
☒	☐	For hierarchical and complex designs, identification of the appropriate level for tests and full reporting of outcomes
☒	☐	Estimates of effect sizes (e.g. Cohen's *d*, Pearson's *r*), indicating how they were calculated

Our web collection on statistics for biologists contains articles on many of the points above.

Software and code

Policy information about availability of computer code

Data collection	Cryo-EM data collection used SerialEM (version 3.8.8) in Titan Krios (Thermo Fisher Scientific). ITC experiments were conducted in a MicroCal PEAQ ITC device (Malvern). Luminescent was read from a SpectraMax M2 luminometer (Molecular Devices). Immunoblotting were imaged using SuperSignal West Pico Plus Chemiluminescent Substrate (Thermo Fisher Scientific) and an iBright FL1000. Fluorescence polarization was measured on a BioTek Synergy Neo2 multimode reader. SEC-MALS was conducted in a FPLC (GE Healthcare) connected with a DAWN HELIOS II MALS detector and an Optilab T-rEX differential refractometer (Wyatt Technology). Mass spectrometry was performed in a UHPLC (Agilent 1290 Infinity II) coupled with a mass spectrometer (Agilent 6470).
Data analysis	Cryo-EM images were analyzed by RELION-3.1, MotionCor2-1.4.2, CTFFIND-4.1.10, cryoSPARC-3.1, ChimeraX 1.2.5, Coot (version 0.9.8), Phenix (version 1.19.2), MolProbity (version 4.5). The binding isotherms of ITC experiment were plotted using MicroCal PEAQ ITC analysis software (Malvern). The luciferase assay, ADP/ATP exchange assay, fluorescence anisotropy, SEC-MALS, Mass spectrometry and quantifications of co-immunoprecipitation were plotted using Prism 8 software (GraphPad).

For manuscripts utilizing custom algorithms or software that are central to the research but not yet described in published literature, software must be made available to editors and reviewers. We strongly encourage code deposition in a community repository (e.g. GitHub). See the Nature Portfolio guidelines for submitting code & software for further information.

Data

Policy information about availability of data

All manuscripts must include a data availability statement. This statement should provide the following information, where applicable:

- Accession codes, unique identifiers, or web links for publicly available datasets
- A description of any restrictions on data availability
- For clinical datasets or third party data, please ensure that the statement adheres to our policy

Cryo-EM 3D composite map of M. tuberculosis DnaK-GrpE complex at 3.7 Å average resolution has been deposited in the Electron Microscopy Data Bank under accession code EMDB-29912. The original map was deposited under accession codes EMDB-29913. Focused map of DnaK SBD domain was deposited under accession codes EMDB-29914. The corresponding atomic model has been deposited in the Protein Data Bank under accession code 8GB3. Source data are provided with this paper.

Research involving human participants, their data, or biological material

Policy information about studies with human participants or human data. See also policy information about sex, gender (identity/presentation), and sexual orientation and race, ethnicity and racism.

Reporting on sex and gender	n/a
Reporting on race, ethnicity, or other socially relevant groupings	n/a
Population characteristics	n/a
Recruitment	n/a
Ethics oversight	n/a

Note that full information on the approval of the study protocol must also be provided in the manuscript.

Field-specific reporting

Please select the one below that is the best fit for your research. If you are not sure, read the appropriate sections before making your selection.

[x] Life sciences [ ] Behavioural & social sciences [ ] Ecological, evolutionary & environmental sciences

For a reference copy of the document with all sections, see nature.com/documents/nr-reporting-summary-flat.pdf

Life sciences study design

All studies must disclose on these points even when the disclosure is negative.

Sample size	The size of the sample for cryo-EM studies were determined by the properties and qualities of the particles and the number of particles in each micrograph. To achieve higher resolution, we usually collect tremendous number of micrographs. For this study, we collected a dataset of 15,720 raw movie micrographs, leading to 9,973,114 raw particles. After 2D classification, a total of 289,063 particles were retained for 3D classification. 240,737 particle were selected after 3D classification. Further 3D refinement resulting in a 3.7Å map. Focused 3D classification and refinement generated a 5Å map of the DnaK SBD domain. The sample size was deemed sufficient when the obtained 3D maps had enough details for atomic model building.
Data exclusions	For the cryo-EM studies, "bad" raw particles of the DnaK-GrpE complex that did not produce 2D class averages or 3D class maps with defined features were excluded after 2D and 3D classifications. This criteria is imperial but is a standard image processing practice in the cryoEM community. For the ITC experiments, data point from the first injection was excluded. The first injection was usually account for a test run and was automatically excluded by the ITC analysis software. For the fluorescence anisotropy assay, we used serial dilution of GrpE protein for the competitive binding to DnaK with nucleotide and substrates. The first data point for the undiluted GrpE usually generated confusing signal, which was likely caused by the protein aggregation due to the very high protein concentration, thus we excluded the first data point.
Replication	For the luciferase assay, ADP/ATP exchange assay, fluorescence anisotropy assay, mass spectrometry and quantifications of co-immunoprecipitation, the reactions were done in triplicate with similar results. For mass spectrometry, For ITC experiment, the reactions were done in duplicate with similar results. For cryo-EM studies, no replicate experiments were performed. Reproducibility resides in the large number of particles used to derived at the final 3D maps or 2D averages. The reliability and the resolution is measured by gold-standard Fourier Shell Correlation. Replication efforts with multiple refinement runs yielded was successful, yielding similar 3D maps.
Randomization	The allocation or selection of 'good' or 'junk' particles are automatically determined by computer program (RELION-3.1 and CryoSPARC-3.1) based on the 2D or 3D templates provided prior running the program.
Blinding	The investigators were not blinded to the specific data points during data collection and analysis, because visual inspection is necessary to ascertain the data quality.

Reporting for specific materials, systems and methods

We require information from authors about some types of materials, experimental systems and methods used in many studies. Here, indicate whether each material, system or method listed is relevant to your study. If you are not sure if a list item applies to your research, read the appropriate section before selecting a response.

Materials & experimental systems

n/a	Involved in the study
☐	☒ Antibodies
☒	☐ Eukaryotic cell lines
☒	☐ Palaeontology and archaeology
☒	☐ Animals and other organisms
☒	☐ Clinical data
☒	☐ Dual use research of concern
☒	☐ Plants

Methods

n/a	Involved in the study
☒	☐ ChIP-seq
☒	☐ Flow cytometry
☒	☐ MRI-based neuroimaging

Antibodies

Antibodies used

For protein and epitope tag detection, the following primary antibodies were used: ALFA (purified nanobody ALFA, 10 mg/ml 1:5000), DnaK (rabbit antisera, 1:10,000), GrpE (rabbit antisera, 1:10,000 PMID: 32996193), mCitrine (Invitrogen, Mouse Anti-GFP monoclonal antibody, 1 mg/ml, 1 : 10,000), and RNAP-β (BioLegend, 8RB13 Mouse Anti-E. coli RNAPβ monoclonal, 1 : 10,000, Cat # 663905).
Secondary antibodies used: anti-VHH-HRP (GenScript, 1:10,000, Cat #A01861-200), anti-mouse-HRP (Invitrogen, 1:5,000, Cat #62-6520), anti-rabbit-HRP (Invitrogen, 1:5,000, Cat # 65-6120).

Validation

The validity of the DnaK and GrpE antibody (rabbit antisera) was demonstrated in the previous publication (ref 65). The nanobody ALFA were validated from a previous publication in ref 63. This paper shows the ALFA-tag is a rationally designed epitope tag that serves a remarkably broad spectrum of applications in life sciences while outperforming established tags like the HA-, FLAG®- or myc-tag. Anti-HRP was used according to the manufacturer's protocols.

Plants

Seed stocks

n/a

Novel plant genotypes

n/a

Authentication

n/a
